# Longitudinal dynamics of the HIV-specific B cell response during intermittent treatment of primary HIV infection

**DOI:** 10.1371/journal.pone.0173577

**Published:** 2017-03-15

**Authors:** Godelieve J. de Bree, Adam K. Wheatley, Rebecca M. Lynch, Madhu Prabhakaran, Marlous L. Grijsen, Jan M. Prins, Stephen D. Schmidt, Richard A. Koup, John R. Mascola, Adrian B. McDermott

**Affiliations:** 1 Department of Internal Medicine, Academic Medical Center, Amsterdam, the Netherlands; 2 Institute for Global Health and Development, University of Amsterdam, Amsterdam, The Netherlands; 3 Vaccine Research Center, National Institute of Allergy and Infectious Diseases, National Institutes of Health, Bethesda, Maryland, United States; CEA, FRANCE

## Abstract

**Background:**

Neutralizing antibodies develop in natural HIV-1 infection. Their development often takes several years and may rely on chronic virus exposure. At the same time recent studies show that treatment early in infection may provide opportunities for immune preservation. However, it is unknown how intermittent treatment in early infection affects development of the humoral immune response over time. We investigate the effect of cART in early HIV infection on the properties of the memory B cell compartment following 6 months of cART or in the absence of treatment. The patients included participated in the Primo-SHM trial where patients with an early HIV-1 infection were randomized to no treatment or treatment for 24 or 60 weeks.

**Methods:**

Primo-SHM trial patients selected for the present study were untreated (n = 23) or treated for 24 weeks (n = 24). Here we investigate memory B cell properties at viral set-point and at a late time point (respectively median 54 and 73 weeks) before (re)-initiation of treatment.

**Results:**

At viral set-point, the memory B cell compartment in treated patients demonstrated significantly lower fractions of antigen-primed, activated, memory B cells (p = 0.006). In contrast to untreated patients, in treated patients the humoral HIV-specific response reached a set point over time. At a transcriptional level, sets of genes that showed enhanced expression in memory B cells at viral setpoint in untreated patients, conversely showed rapid increase of expression of the same genes in treated patients at the late time point.

**Conclusion:**

These data suggest that, although the memory B cell compartment is phenotypically preserved until viral setpoint after treatment interruption, the development of the HIV-specific antibody response may benefit from exposure to HIV. The effect of viral exposure on B cell properties is also reflected by longitudinal changes in transcriptional profile in memory B cells over time in early treated patients.

## Introduction

Patients that start treatment in the early phase of infection are currently seen as candidates for therapeutic interventions aiming to achieve post-treatment viral remission, such as therapeutic vaccination. The very early reduction in viral reservoir in these early treated patients may be key for the potential preservation of HIV-specific immune responses. From this perspective, well-equipped T and B cells that control virus replication after cART interruption are considered to be very important. Therefore, the identification of immune parameters associated with preservation of the memory response during HIV infection is important to providing clues for the development of therapies needed to achieve post-treatment viral control. In the present study we focus on the development of the humoral immune response in a cohort of patients that were intermittently treated during early infection.

The development of a broadly neutralizing antibody (bNab) response is a key component of an effective protective HIV-specific immune response and a target for vaccine development [1][2][3][4]. Furthermore, bNabs limit viral rebound after structured treatment interruptions [5] and reduce viremia in non-human primates [6][7] and humans [8]. However, only 20% of HIV infected individuals develop bNabs that can neutralize greater than 80% of genetically diverse viruses [9][10][11]. Although viral exposure is an important driving factor for the formation of bNab, the precise mechanisms leading to development and maintenance of bNab remain to be elucidated. Furthermore, how treatment initiated in early infection affects the development of HIV-specific humoral immune response once treatment is interrupted, is still unknown. Insight herein may help the design of therapeutic interventions.

The generation of long lasting protective humoral immunity requires the elicitation of neutralizing antibodies secreted from long lived plasma cells, in addition to the establishment of a pool of antigen experienced memory B cells [12]. However, the homeostasis of the memory B cell compartment becomes perturbed during the natural course of HIV infection. This perturbation consists of an increased fraction of activated memory cells, plasmablasts and exhausted B cells at the expense of long lived plasma cells [13][14]. In acute and chronic viremic HIV-infected individuals, envelope-specific, class-switched IgG expressing B cells, are enriched in these activated memory and in resting memory B cell subsets and found at lower frequencies in the tissue-like and intermediate memory B cell subsets [15]. At a functional level, HIV infection induces hyperglobulinemia and drives expansion of cells with an exhausted B cell phenotype (CD19^pos^CD27^neg^CD21^neg^FCRL4^pos^). The induction of exhausted B cells is also characteristic of other chronic infections and some autoimmune disorders [16][17][18]. Paradoxically, the same factors that drive B cell exhaustion are also related to the development of neutralization breadth. In this context, the duration of infection, virus load and viral diversity have been linked to the development of bNabs [19][20][21][22][23]. These studies suggest that a balance may exist between viral exposure, perturbation of the B cell response and the development and maturation of Nab. A key question is how this balance is driven by the modulation of viral exposure and how it affects the properties of the B cell compartment.

Studies in patients with chronic HIV-1 infection show that reduction in viral load by combination anti-retroviral therapy (cART) leads to a reduction in polyclonal B cell responses but partial restoration of perturbed B cell subpopulations [24][25][26][27][28]. The effect of cART upon the restoration of memory B cell responses was more evident when cART was initiated early in the course of infection. This leads to improved *in vitro* memory B cell responses to HIV envelope (gp120) and influenza, compared to individuals who started cART during chronic infection [29]. Therefore, in the present study we explore how intermittent intervention with cART during the early phase of infection to promptly contain viral replication affects the memory B cell compartment. Moreover, it is not known how phenotypic alterations of memory B cell subsets relate to the generation of Nab responses after interruption of cART that has been initiated in early infection. Here we were able to address the effect of early cART upon the properties of the memory B cell compartment following 6 months of cART or in the absence of treatment. The Primo-SHM trial lends itself to perform memory B cell analysis in patients who were or were not treated during early infection [30]. We explore memory B cell phenotype, transcriptional profile, function (HIV-envelope-specific antibody titers and neutralization breadth) at viral set point (36 weeks after diagnosis in untreated patients or 36 weeks after treatment interruption in the treated patients) and at the latest available time point before (re)-start of therapy in case of decreasing CD4 counts.

## Materials and methods

Please see supplementary materials (S1 File) for details of the materials and methods used in this study.

### Study subjects

**Fig 1:**
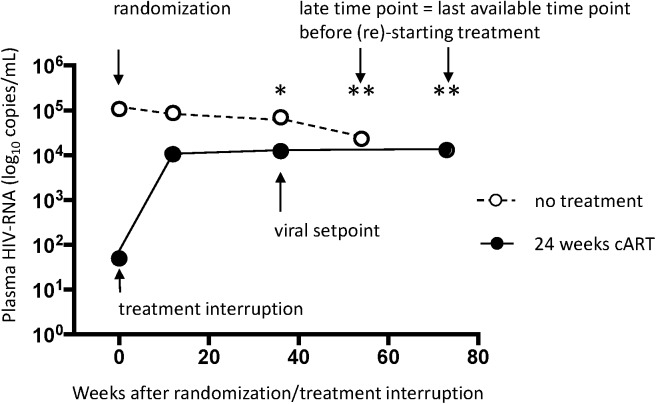
Plasma viral load after randomization / treatment interruption in untreated and 24 weeks treated patients. The patients depicted consist of a subgroup of the initial Primo-SHM trial [30]. The graph shows the median viral load over time. The time points that were selected for analyses in the present study are viral setpoint (defined as 36 weeks after randomization / treatment interruption) (*) and the latest available time point before (re)-start of treatment (median weeks for each group) (**) (see also Table 1).

The study population consisted of patients who were enrolled in the Primo-SHM study [30]. Patients included in the Primo-SHM study were over 18 years and had laboratory evidence of PHI infection, defined as a negative or indeterminate Western blot in combination with detectable plasma HIV-1 RNA (Fiebig stage I–IV) or, in case of a positive Western blot, a documented negative HIV screening test in the previous 180 days (Fiebig stage V–VI [31]). In the Primo-SHM study patients were randomized to ‘no-treatment’ or ‘short term’ treatment for 24 weeks or 60 weeks. Briefly, the results of the Primo-SHM study were as follows. Patients randomized to short- term treatment had a significantly lower viral set-point (4.0 (standard deviation (sd) 1.0 log10 copies/ml (24 weeks arm) and 4.3 (sd 0.9) log10 copies/ml (60 weeks arm) compared to the no-treatment arm (4.8 (sd 0.6) log10 copies/ml) (p = 0.001). The median total time off therapy in the untreated group was 0.7 (95% CI 0.0–1.8) years compared to 3.0 (1.9–4.2) years in the 24 weeks treated group and 1.8 in the 60 weeks treated group (p = 0.001) and [30]. The patients selected for the present study were those of whom plasma samples were available. Furthermore, given the similar clinical outcome in the two treatment arms, patients receiving 24 weeks of treatment were included for the analyses. In the present study patient samples obtained at viral set-point (defined as plasma viral load (pVL) at 36 weeks after randomization in untreated patients and 36 weeks after treatment interruption in the early treated patients to allow for stabilization of the pVL) and the last available time point before (re-) start of cART were chosen to allow for analysis of neutralizing antibodies (Fig 1). Short term treated patients are hereafter referred to as the “treated” patient group and patients that received no treatment to “untreated” patient group.

**Table 1:** Characteristics of study participants.

	*untreated*	*treated*	*p-value*
***Number of patients***	23	24	
***Age***	42 (26–56) ^a^	41 (25–60)	0.89
*Viral setpoint*			
***CD4pos T cell count (cells/uL)***	340 (210–610)	600 (230–1510)	0.0001
***HIV RNA (copies/mL)***	7x10^5^(3,3x10^3^-1,2x10^6^)	1,2x10^4^(53–2,8x10^5^)	0.004
*Late time point*			
***CD4pos T cell count (cells/uL)***	340 (170–730)	360 (190–670)	0.56
***HIV RNA (copies/mL)***	2,3x10^4^ (319–5,3x10^5^)	1,3x10^4^ (268–2,5x10^5^)	0.84
***weeks between viral setpoint and late sampling time point***	54 (14–183)	73 (24–301)	

^a^ In the table median values and ranges are given.

The Primo-SHM trial was prior to initiation approved by the Medical Ethics Committee of the Academic Medical Center. The informed consent included consent for participation in the trial as well as for viro-immunological analyses. All patients gave written informed consent.

### Flow cytometry

PBMC were obtained by density-gradient centrifugation and cryopreserved prior to analysis. Multicolor flow cytometry was performed by using standard protocols using the following antibodies: CD3 (QD655), CD14 (QD800), CD19 (ECD), CD20 (allophycocyanin [APC]-Cy7), CD21 (Cy5PE), CD27 (QD705), CD38 (Alexa680), and IgG (BrilliantViolet421), IgD (Cy7PE), IgA (APC) and IgM (Cy55PerCP) and CXCR5 (Alexa488). Antibody labeling was performed at room temperature for 30 min. The data were collected on a LSRII flow cytometer using FACSDiva software (BD Biosciences). Color compensation was performed using single-stained samples for each fluorochrome used in addition to an unstained control. The data were further analyzed by using FlowJo software (TreeStar, Cupertino, CA). Flow cytometric cell sorting was performed on a 20-parameter FACSAria (BD), running FACSDiVa software (version 6.1.3 (BD)). HIV-specific memory B cells were identified by staining with a trimeric PE labeled YU2 gp140-foldon probe. Influenza-specific memory B cells were identified by staining with an H1-APC labeled probe.

### Gene expression profile

B cell subsets were sorted at 100 cells per well in duplicates for pooled cell (nanoarray) dynamic RT-qPCR array (Fluidigm Biomark) and gene expression profiles were quantified using a panel of 96 gene-specific primers as previously described [32]. Results were analyzed using JMP version 10. For each of the genes in the panel, two ratios were calculated as follows: the first ratio denoted as ‘temporal variation’ was calculated as the ratio of difference in gene expression occurring over time from viral setpoint to the late time point in the treated group, to the difference in gene expression occurring over time from the viral setpoint to the late time point in the untreated group. The second ratio denoted as ‘inter-group’ variation was calculated as the ratio of the difference in gene expression observed at viral setpoint between the two groups to the difference in gene expression observed at the late time point between the two groups. Statistical significance was computed for gene expression differences over time observed within each group and between the two groups at each time point using one-tailed and two-tailed tests. P values ≤ 0.05 on the two-tailed t test were considered significant. Furthermore, genes were mapped to pathways using KEGG Mapper.

### Probes for the detection of envelope-specific antibodies

To detect serum antibodies directed towards total envelope, gp 41 (membrane proximal region (MPER)) and CD4 binding site (CD4bs), well characterized recombinant proteins were used: Total envelope antibodies were detected using YU2 gp140-foldon [33]. CD4bs reactive antibodies were measured by using a resurfaced stabilized gp120 core (RSC3) as described previously [34][35]. The RSC3 probe is a resurfaced version of the stabilized core of gp120 (HXB2Ds12F123) [36]. MPER reactive antibodies were detected by reactivity against 10E8 peptide epitope [37].

### HIV-1 protein binding and neutralization assays

To evaluate the ability of patients to neutralize virus, a 6-virus panel of heterologous pseudo-viruses was constructed. The panel contained a selection of tier 1 and 2 viruses from 3 clades (clade A: DJ263 (tier 1b), Q168.a2 (tier 2); clade B: SF162 (tier 1a), JRFL (tier 2); clade C: ZM109.4 (tier 1b), DU156.12 (tier 2)). Serum antibodies against HIV-1 gp140, CD4 binding site and MPER were measured as previously described [38]. Based on background levels in sera from uninfected donors and titers against a control pseudovirus using murine leukemia virus Env, the cutoff for neutralization was set at an ID50 of 100.

### Statistical analysis

Between-group analyses were performed using the nonparametric Mann-Whitney *U* test. Comparisons within groups were made using the Friedman test. If this yielded a significant result, further pairwise comparisons within the group were made using the Wilcoxon matched-pairs signed ranks test. Two-sided testing was done; *P* < .05 was considered to be statistically significant. The software used for statistical analysis was IBM SPSS statistics (version 22).

## Results

### Patient characteristics

The Primo-SHM study patients diagnosed in early infection were randomized between two arms: those that did not receive cART at diagnosis (untreated group) and those who received 24 weeks of cART (treated group). The patients that were analyzed in the present study are a subgroup of the total Primo-SHM trial and consist of patients of whom samples were available. The two time points that were selected for the analyses in the present study were viral set point (36 weeks after randomization or 36 weeks after treatment interruption) and the latest available time point prior to (re-) start of cART (hereafter named as late time point) (median 54 weeks (range 21–183) after inclusion in the study in untreated patients and median 73 weeks (range 24–301) after treatment interruption in treated patients (Fig 1 and Table 1).

### Changes in memory B cell compartment in treated patients until viral set point after treatment interruption

**Fig 2:**
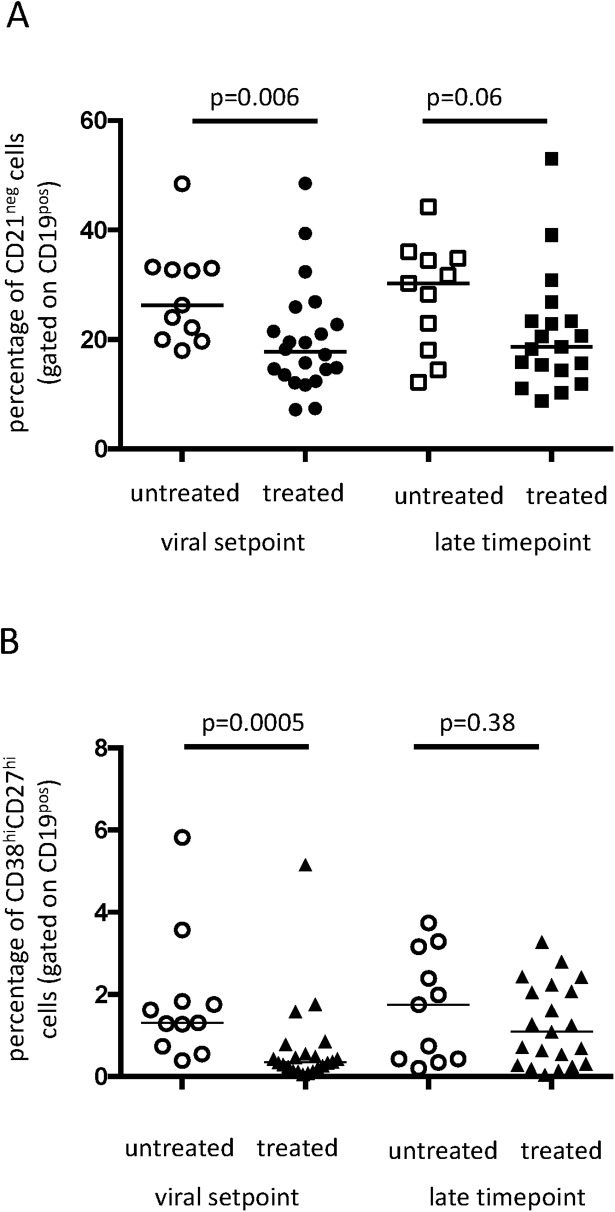
**Treated patients show A: a smaller fraction of antigen-primed, CD21**^**neg**^**, B cells at viral setpoint as well as B: a smaller fraction of plasmablasts at viral setpoint.** Horizontal bars represent median values and statistical significance was calculated using the nonparametric Mann-Whitney *U* test.

**Fig 3:**
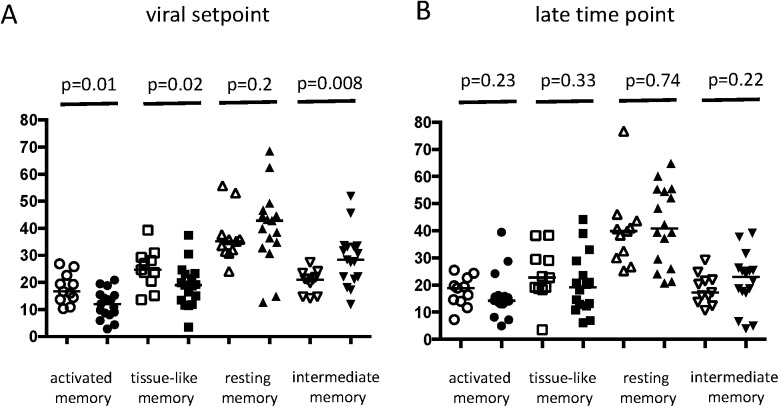
**Differences in phenotype of the B cell compartment at viral setpoint (A) and the late time point (B) between untreated (empty symbols) and treated (filled symbols) patients.** Scatterplots show the frequency of memory B cells divided by subset. Horizontal bars represent median values and statistical significance was calculated using the nonparametric Mann-Whitney *U* test. Open symbols represent untreated patients and closed symbols represent treated patients. On the y-axis percentages of cells are depicted gated on aqua^neg^CD19^pos^IgG^pos^ cells

We first set out to characterize the phenotype of B cells with respect to known B cell differentiation markers. Gating strategies are shown in S1 Fig. Expression of CD21 can be used to discern antigen experienced (immature/transitional B cells, tissue-like or activated memory) B cells and an increase in CD21^neg^ B cells is associated with HIV-induced B cell expansion [39]. In the treated patient group, at viral set point, the fraction of CD21^neg^ cells was significantly higher compared to untreated patients (p = 0.006). At the late time point this difference persisted, albeit not significant (p = 0.06) (Fig 2A). Furthermore, plasmablasts (PB) (CD19^pos^CD27^hi^CD38^hi^) were significantly higher in untreated patients at viral set point (p = 0.0005) compared to treated patients (p = 0.38) (Fig 2B). Next we characterized the distribution of subtypes of memory B cells based on CD21 and CD27 within IgG^pos^ memory fraction: activated memory (AM) B cells (CD21^neg^CD27^pos^), intermediate memory (IM) B cells (CD21^pos^CD27^neg^), resting memory (RM) B cells (CD21^pos^CD27^pos^) and tissue-like memory (TLM) B cells. Treated patients showed at viral setpoint a significantly lower percentage of AM B cells (CD21^neg^CD27^pos^) (p = 0.01), TLM B cells (CD21^neg^CD27^neg^) (p = 0.02) and IM B cells (CD21^pos^CD27^neg^) (p = 0.008) (Fig 3A), as compared to untreated patients. At the late time point (54 weeks (median) after viral setpoint in the untreated group and 73 weeks (median) in treated patients) these differences in memory B cell subsets were no longer apparent (Fig 3B). When interpreting the given p-values one should realize that shifts in B cell subsets are interconnected. These data suggest that intermittent treatment during early infection is associated with prolonged phenotypic changes in the memory B cell compartment in the peripheral blood until viral set point. However, these phenotypic changes are transiently lost as time progresses, CD4 counts decline and virus rebounds [30].

**Fig 4:**
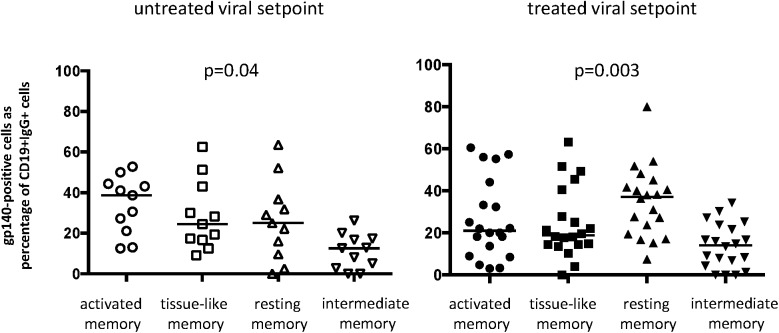
The phenotype of HIV gp-140-specific B cells shows a shift towards a predominance of resting memory B cell phenotype in treated patients. Scatterplots show the distribution of gp140 positive memory within IgG^pos^CD19^pos^ B cells (RM: resting memory; IM: intermediate memory; AM: activated memory; TL: tissue like memory) in untreated (left) and treated (right) patients at viral setpoint. Horizontal bars represent median values. On the y-axis percentages of gp140^pos^ cells expressed as percentage of IgG^pos^CD19^pos^ cells are depicted. P values show the significance level calculated by Friedman test. Further testing for differences between B cell subsets in each group was performed using Wilcoxon matched-pairs signed ranks test and shows the following significance levels. In summary for treated patients: RM compared to IM p<0.001; RM compared to AM p = 0.16; RM compared to TLM p = 0.18. In summary for untreated patients: RM compared to IM p = 0.08; RM compared to AM p = 0.4; RM compared to TLM p = 0.8.

In order to investigate the phenotype of envelope-specific B cells at both time-points, a YU2 gp140 probe, containing a labeled trimerized envelope complex, was used. As a control for specificity of the YU2 gp140 probe, we performed combined staining with an influenza probe (S1 Fig). The overall frequency of gp140-specific IgG^pos^ B cells between groups at viral set-point were comparable ((untreated group: gp140-positive cells as percentage of IgG^pos^ B cells: median 0.81 (range 0.15–2.09)); treated group: gp140-positive cells as percentage of IgG^pos^ B cells: median 0.62 (range 0.12–2.1)(p = 0.71). Next, within the gp140-positive IgG^pos^ B cell fraction, the phenotypic distribution of the B cells was determined. In treated patients, the phenotype of B cells tends to shift towards a higher frequency of RM, whereas in untreated patients this shift did not reach statistical significance (Fig 4).

### Longitudinal changes in transcriptional profile of memory B cells

**Fig 5:**
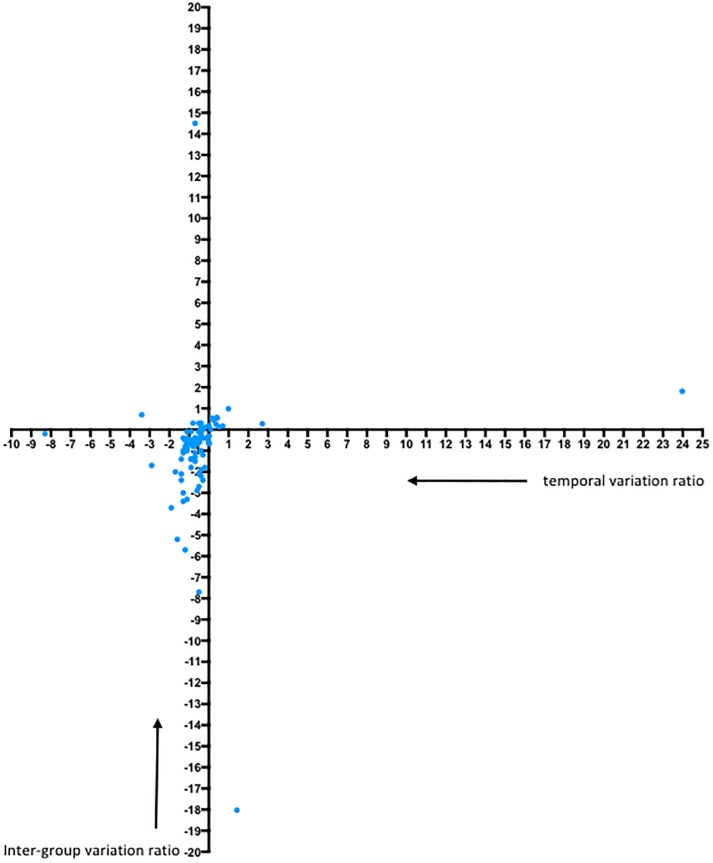
Dynamic shifts in gene expression occurring within each group with time was quantified as a ratio between temporal gene expression differences observed in the treated group to that of the untreated group, for each of the 92 genes. This temporal variation ratio was taken as the x-coordinate. Differences in gene expression observed between the two groups at each time point was quantified as a ratio between differences observed between the two groups at viral setpoint to that observed at the late time point, for each of the 92 genes. This inter-group variation ratio was taken as the y-coordinate. This graphs shows the distribution of the 92 genes based on the two calculated ratios. The polarization towards the double negative quadrant indicates that temporal variation occurring with time within each group occurs in opposite directions; and that the inter-group variation observed between the two groups at the viral setpoint and the late time point are in opposite directions. In addition, for 65 genes temporal variation ratio was <1 indicating that the changes in gene expression accrued with time in the untreated group was greater than changes accrued with time in the treated group. Also, for 59 genes inter-group variation ratio was <1 indicating that differences in gene expression observed between the two groups were greater at the late time point compared to those observed at the early time point.

The phenotypic characterization of the B cell compartment in our patient cohort suggests that the intermittent treatment of early infection results in a relative redistribution of B cell fractions, even until 36 weeks after treatment interruption (viral set point). To investigate how early treatment affects the transcriptional profile in memory B cells (IgG^po*s*^CD21^pos^CD27^pos^) over time, after treatment interruption, a 96-gene panel consisting of genes involved in cell signaling, adhesion, differentiation, activation, maturation, proliferation and trafficking was developed. At viral setpoint and at the late time point a multiplexed qPCR assay was run in both groups. Four of the genes in the panel did not yield any expression (AICDA, B4GALT1, PAX5, TCL1A). In order to quantify dynamic shifts in gene expression levels between the two groups at viral set point and the late time point, two ratios were computed for each of the remaining 92 genes (see methods section and S1 Table): the temporal variation ratio to quantify gene expression changes occurring over time within each group and the inter-group variation ratio to quantify differences in gene expression observed between the two groups at each time point. In Fig 5 the results are displayed for temporal variation (x-axis) and inter group variation (y-axis). The analysis showed that the data points of 70 genes (76%) localized to lower left quadrant and 11 genes (12%) to the upper right quadrant (Fig 5). This indicates that temporal variation within each of the groups occurred respectively in 76% in opposite and in 12% in the same direction as did the inter-group variation between the two groups at each time point.

To investigate if the of the size temporal variation (= changes over time) differed between the two groups, the temporal variation ratio was calculated. For the majority (65 of the 92) of genes (71%), the temporal variation ratio was less than 1, indicating that temporal variation from viral setpoint to the late time point within the untreated group was greater than that in the treated group. To investigate the magnitude of differences between groups at viral set point compared to the late time point, the inter-group variation was calculated. This analysis showed that for 59 of the 92 genes (64%), the inter-group variation ratio was less than 1, indicating that differences in gene expression between the untreated and treated groups at the late time point were greater than the observed differences at viral setpoint. 81% of those 59 genes were also a subset of the 65 genes that accrued more gene expression changes over time in the untreated group compared to the treated group.

In the untreated group, 59 genes (64%) showed significantly higher gene expression at viral setpoint compared to the late time point, while in the treated group, only 5 genes (5%) showed significantly higher gene expression at viral set point compared to the late time point. Of these 59 genes with a higher expression at viral set point in the untreated group, 41 genes (70%) were also found to be significantly upregulated with time in the treated group. These genes constitute 89% of all the genes that had significantly higher expression at the late time point. This could again be a reflection of increasing viral loads in the treated group that drives the B cell compartment towards a state comparable to that of the untreated group at viral setpoint.

### Untreated patients mount a significantly better envelope-specific antibody response over time compared to treated patients

**Fig 6:**
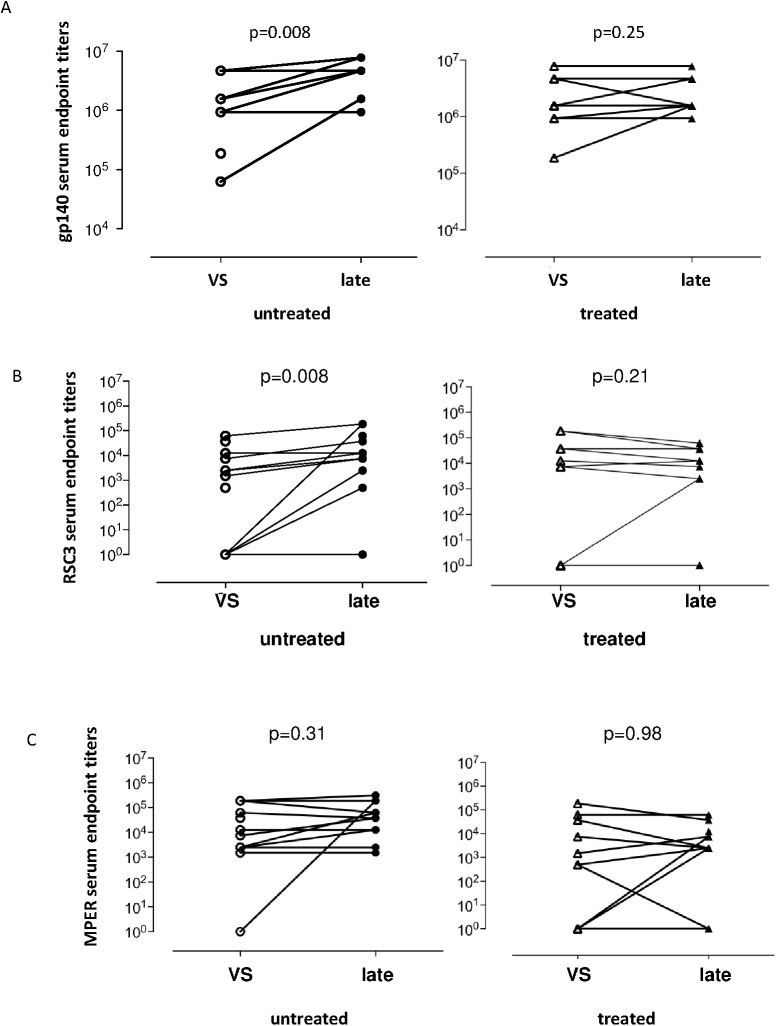
**Scatterplots showing the serum endpoint titers of antibodies** directed against gp140 (**A**), CD4 binding site (CD4bs) (resurfaced stabilized gp120 core (RSC3))(**B**) and membrane proximal region (MPER) (**C**) in untreated (left panels) and treated (right panels) patients at viral set point (VS) and at the late time point (late). Statistical significance was calculated with nonparametric Wilcoxon matched-pairs signed-ranks test.

Finally, the effect of early modulation of viral exposure on the generation of the HIV-specific antibody response was investigated. Both patient groups showed a comparable increase in number of viruses neutralized over time (S2 Fig). Next, we determined the serum antibody titer of total gp140 specific antibodies. Although both patient groups had comparable levels of binding anti-gp140 antibody titers, untreated patients showed a significant increase in total binding antibody titers over time compared to treated patients (Fig 6A). Env-specific antibodies can be subdivided into categories based on the epitopes of the envelope to which they bind. In order to investigate the longitudinal dynamics of the development of envelope-specific antibodies, antibody binding titers against CD4 binding site (CD4bs), resurfaced stabilized gp120 core (RSC3) and membrane proximal region (MPER) were determined [40][41][37]. At viral set point, 14 of 22 patients in the untreated group (64%) had detectable antibodies against CD4bs, versus 8 of 16 patients in the treated group (50%). In contrast to treated patients, untreated patients showed a significant increase in CD4bs titer over time (p = 0.21 and p = 0.008, respectively) (Fig 6B). MPER binding antibodies were detectable in all but one patient in the untreated group and 13 of 16 patients in the treated group at viral set point. MPER antibody binding titers were stable over time in both groups (p = 0.31 untreated patients and p = 0.98 treated patients) (Fig 6C). These data suggest that untreated patients mount an enhanced envelope antibody binding titer over time compared to patients treated in the early phase of infection.

## Discussion

In the present study we characterized the effect of intermittent cART intervention during the early phase of infection on the humoral immune response by prompt containment of viral replication. The Primo-SHM study, where patients were temporarily treated during early infection, allowed us to address this subject. We showed a shift in the B cell subpopulations towards phenotypically exhausted and activated subsets at viral setpoint in untreated individuals compared to individuals given cART during early infection. These findings suggest that early reduction of viral load phenotypically preserves the B cell compartment for periods after treatment interruption. However, the predominance of this activated and exhausted B cell compartment in untreated patients did not preclude the development of significantly increasing levels of envelope (CD4 binding site) -specific antibodies over time.

Earlier studies showed that shifts in B cell subpopulations occur early after infection, within 6 months after seroconversion, and that the lowering of viral load by cART is associated with less activated and exhausted B cells [28]. In our study, we observed fewer activated and antigen-primed B cells in the periphery in treated patients compared to untreated, and these numbers were maintained for a median of 24 weeks after structured treatment interruption. B cell dysfunction in HIV can either be the result of direct binding of virions to the CD21 molecule or through immune activation and / or reduced T follicular helper cell function [13][42][43]. Although our study does not provide a mechanistic insight in how cART interferes with HIV induced B cell changes, the findings in our study in which viral load is modulated by early treatment, point to a role of viral exposure (reflected by height of viral load and duration of infection). Furthermore, plasmablasts have a profile characterized by increased expression of activation markers and enhanced proliferative capacity that is normalized by lower levels of virus [44]. The frequency of plasmablasts in early HIV infection (with high plasma viral loads) was higher in early compared to chronic infection. This is indicative of a role in viral load burden driving the size of this population [45]. In our patient group we did not observe a direct correlation between viral load at setpoint and the phenotypic distribution of B cell subsets (data not shown), possibly due to the rather small number of patients. The observation that plasmablast frequencies in treated patients in our study increased at the late time point, when virus rebounded during treatment interruption to levels comparable to untreated individuals, is suggestive for the role of viral exposure. Furthermore, in our study we observed a tendency towards a shift of gp140-specific B cells towards the resting memory B cell fraction in treated patients. Although we have to be precautious in drawing firm conclusions given the smaller number of untreated patients compared to treated patients, these data suggest that early treatment may lead to a relative maintenance of RM phenotype within envelope-specific IgG^pos^ B cell fraction.

In addition to the described changes in B cell subset distribution, we used transcriptional profiling to investigate differences within memory B cells between groups and over time. It was found that changes in transcriptional profile were more profound in the untreated group when compared to treated patients. Furthermore, there was a large overlap between genes that were differentially expressed at viral setpoint and at the late time point. This leads to the speculation that the greater differential observed in gene expression levels between the two groups at the late time point might largely be a consequence of the sustained gene expression changes occurring over time in the untreated group due to greater viral exposure. These findings suggest that indeed viral exposure may drive these changes in transcriptional profile in untreated patients and that these are limited by early treatment. These findings suggest that early events in B cell priming may lead to genetic imprinting that is maintained over the course of infection and results in decelerated gene expression changes over time in the treated group. This area of our study warrants further investigation using next generation sequencing to elucidate pathways and mechanisms for potential therapeutic intervention. A caveat in our study is that it relies on peripheral blood evaluation of B cells, and the distribution and mechanisms that underpin the homeostatic maintenance of B cell subsets may differ significantly in secondary lymphoid tissues where humoral responses are initiated.

From perspective of viral factors that drive the formation of neutralizing antibodies, the height of viral load as well as diversification of the virus may play a role. The development of neutralization breadth during acute infection is associated with a high viral load during acute infection [46] and early cART plays a limiting role on viral diversification [47]. An unanswered question thus far is how changes in B cell compartment by early lowering of viral load can be linked to the development of the neutralizing antibody response. The temporal modification of viral load early in the course of infection in the Primo-SHM trial allowed us to address this issue. Neutralizing antibodies are directed against the surface exposed envelope spike that consists of the trimeric gp120 molecule and the transmembrane gp41 molecule [48]. Many antibodies target gp120 and gp41, however only a small number bind to epitopes on the envelope spike that are broadly reactive and can neutralize the virus. The development of the antibody response occurs in a sequential manner whereby the first to appear are non-neutralizing class-switched antibodies [49] including antibodies targeting gp41. In the gp41 molecule, the membrane proximal region of the envelope stalk (MPER) is the site that is target for binding of neutralizing antibodies. Our finding that the increase in MPER-specific antibodies was comparable between untreated and treated patients can be a reflection of very early priming, before start of treatment, of MPER antibodies producing B cells [50]. Much later, in rare cases, broadly neutralizing antibodies develop directed against conserved epitopes on the envelope [51][52][53]. Our observation that the untreated patients, in contrast to the treated patients, developed a significant increase in antibody titers against total envelope and CD4 binding site suggests that it may be the high antigen load that drives this process and that antigenically activated memory B cell subsets may be needed for the generation of this response. Taken together, the enhanced antibody response, in combination with the presence of an exhausted B cell compartment in untreated infected patients as compared to patients that were treated during early infection, illustrate the key paradox in HIV. Our data indicate that at least a certain extent of viral exposure is required for the selection of memory B cells that would give rise to a neutralizing antibody response. Currently ongoing post-treatment control cohort studies focus on patients that start treatment very early after infection. Recent studies showed that the antibody response in patients that start in the earliest phase of acute infection in some cases not even develops [54]. Together with our data this study indicates that at least a certain extent of viral exposure is required for the selection of memory B cells that would give rise to a neutralizing antibody response. Therefore, the design of therapeutic vaccine interventions aiming to achieve post-treatment control by induction of memory responses should take into account that the development of inducible HIV-specific memory may lag in acutely treated individuals.

## Supporting information

S1 File File with description of the details of the materials and methods used in this study. (DOCX)

S1 Fig Gating strategies for B cell subsets. B cells isolated form peripheral blood mononuclear cells were stained for CD19, IgG, CD21, CD27, CD38 and aqua as marker for cell viability. Gates were set on aqua negative, CD19 positive cells. Plasmablasts are defined as CD27^++^CD38^++^, gated on CD19^pos^aqua^neg^, subsets of memory B cells are gated on CD19^pos^aqua^neg^IgG^pos^ and further defined based on expression of CD21 and CD27.
(TIFF)

S2 Fig Neutralization breadth against 6 pseudo viruses at viral set point (VS) and at the late time point (late). For reciprocal serum ID50 values a cutoff of 30 was used. Horizontal bars represent median values.
(TIFF)

S1 Table List of all the genes and their functions that showed statistically significant changes with time within each group and / or statistically significant differences between the 2 groups at one of the two time points (viral set point or late time point). The direction of change is indicated by color: genes shown in red are significantly down regulated with time and genes indicated in green are significantly up regulated with time within a particular group; genes indicated in purple had significantly higher expression in the untreated group and genes indicated in blue had significantly higher expression in the treatment-interrupted group at a particular time point. P values ≤ 0.05 on the two-tailed t test were considered significant.
(DOCX)
