# Supplementary material for: Longitudinal dynamics of the HIV-specific B cell response during intermittent treatment of primary HIV infection
Source: PLoS One. 2017 Mar 15;12(3):e0173577. doi: 10.1371/journal.pone.0173577 (PMC5351995; doi:10.1371/journal.pone.0173577)
Supplement: S1 File — (DOCX) [file pone.0173577.s001.docx]

**Supplementary Methods**

**HIV-1 protein binding assay** ELISAs were performed as previously described (37). Briefly, 96-well ELISA plates were coated with 2 ug/ml of the specified recombinant protein in phosphate-buffered saline (PBS) overnight at 4°C. The following day, the plates were blocked with B3T buffer (150 mM NaCl, 50 mM Tris-HCl, 1 mM EDTA, 3.3% fetal bovine serum, 2% bovine albumin, 0.07% Tween 20) and incubated with 4-fold serial dilutions of heat-inactivated sera starting at a dilution of 1:100, followed by peroxidase-conjugated goat anti-human IgG antibody (Jackson ImmunoResearch). All incubations were for 1 h at 37°C, and all volumes were 100 ul, except for blocking, which was 200 ul. The plates were washed between incubations with 0.1% Tween 20 in PBS, detected using SureBlue TMB substrate (Kirkegaard & Perry Laboratories), and subsequently read at 450 nm. Endpoint titers were calculated based on the final reciprocal serum dilution with a background-corrected optical density (OD) greater than or equal to 0.1. Sera were considered reactive when the reciprocal endpoint titer was greater than 200.

**Neutralization assay** Serum samples from untreated patients and 24 weeks treated patients were used. Thawed patient serum was heat-inactivated at 56°C for 30 min prior to assay. HIV-1 neutralization was measured using a single round of infection by Env pseudoviruses and Tzm-bl target cells as previously described (38). Pseudoviruses (multiplicity of infection [MOI], approximately 0.1) were added for a 30-min incubation. Tzm-bl cells were added at a concentration of 10^4^ per well, and the single- round infection proceeded for 48 h. All incubations were at 37°C, and each infection was performed in duplicate wells of a 96-well flat-bottom culture plate. Neutralization curves were fitted by nonlinear regression using a four-parameter hill slope equation programmed into JMP statistical software (JMP 5.1; SAS Institute Inc., Cary, NC). The 50% inhibitory dilutions (ID50s) were reported as the reciprocal serum dilutions required to inhibit infection by 50%. Based on background levels in sera from uninfected donors and titers against a control pseudovirus using murine leukemia virus Env, the cutoff for neutralization was set at an ID50 of 100.

**Gene expression profile** B cell subsets were sorted at 100 cells per well in duplicates and multiplexed q-PCR was carried out using the 96.96 dynamic array microfluidic chip for gene expression (Fluidigm; Cat# BMK-M-96.96) on the Biomark system as previously described (31), using a panel of 96 gene-specific primers. Results were analyzed using JMP version 12 to identify genes that were differentially expressed between groups 1 and 2 at viral setpoint and the late time point. Ingenuity Pathway Analysis (IPA) version 2016 (Qiagen) was then used to identify pathway enrichments amongst these differentially expressed genes to derive at gene interaction maps. Briefly, the canonical pathway analysis tool was used to query the list of genes that were differentially expressed between groups 1 and 2 at the two time points, against the library of canonical pathways stored in the IPA database to identify pathways most represented by the genes. The percent representation in the pathway was calculated as a ratio between the number of genes in the input dataset identified to be involved in a particular canonical pathway and the total number of genes documented in IPA to be involved in that pathway. A p value was generated by Fisher’s exact test for each pathway association to estimate the probability that the mapping of the genes from the input dataset to the canonical pathway is due to chance alone. The p values of all associations made were below 10^-5^.
