# Supplementary figures and images for: Longitudinal dynamics of the HIV-specific B cell response during intermittent treatment of primary HIV infection
Source: PLoS One. 2017 Mar 15;12(3):e0173577. doi: 10.1371/journal.pone.0173577 (PMC5351995; doi:10.1371/journal.pone.0173577)

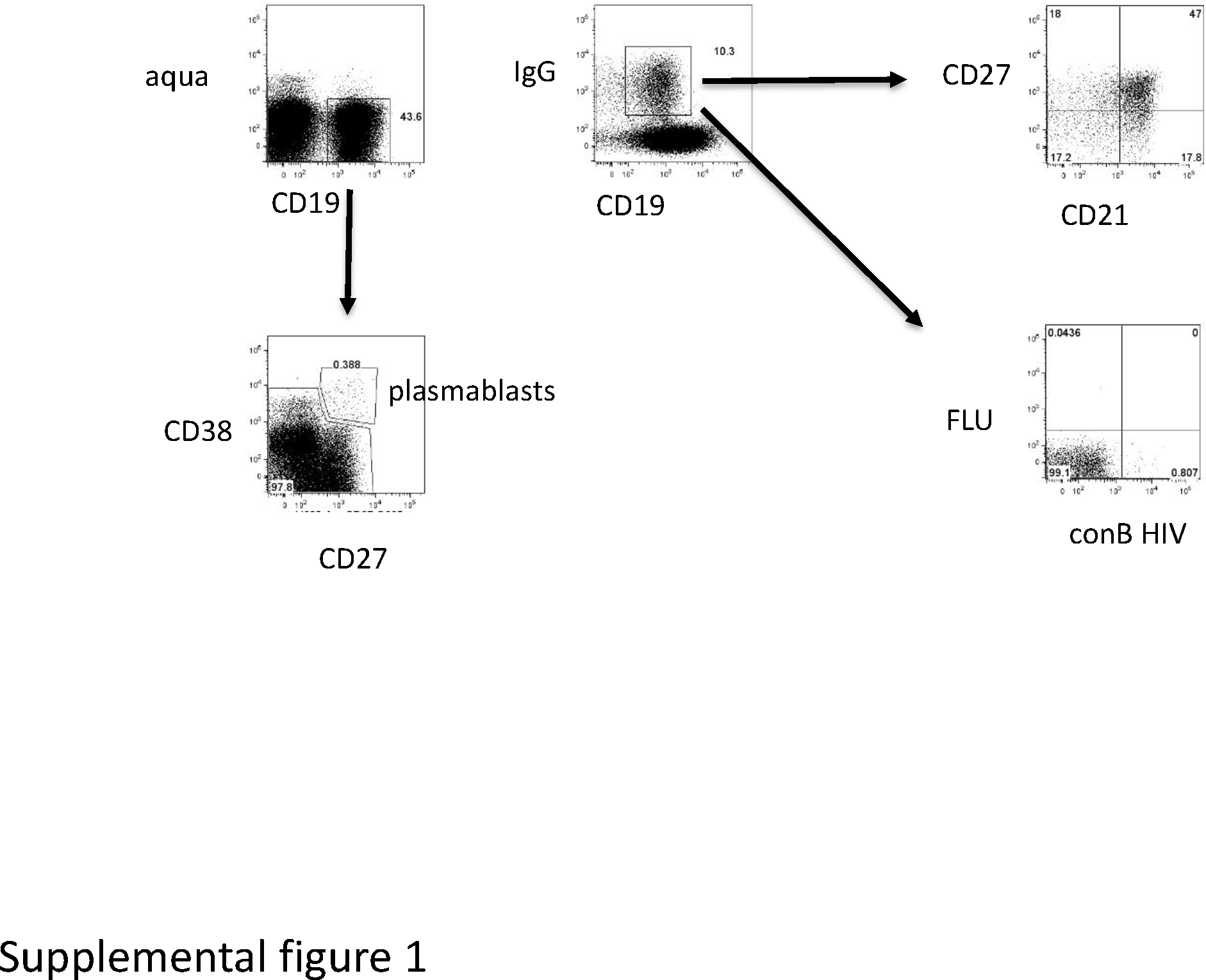

Supplement: S1 Fig — B cells isolated form peripheral blood mononuclear cells were stained for CD19, IgG, CD21, CD27, CD38 and aqua as marker for cell viability. Gates were set on aqua negative, CD19 positive cells. Plasmablasts are defined as CD27++CD38++, gated on CD19posaquaneg, subsets of memory B cells are gated on CD19posaquanegIgGpos and further defined based on expression of CD21 and CD27. (TIFF) [file pone.0173577.s002.tiff]

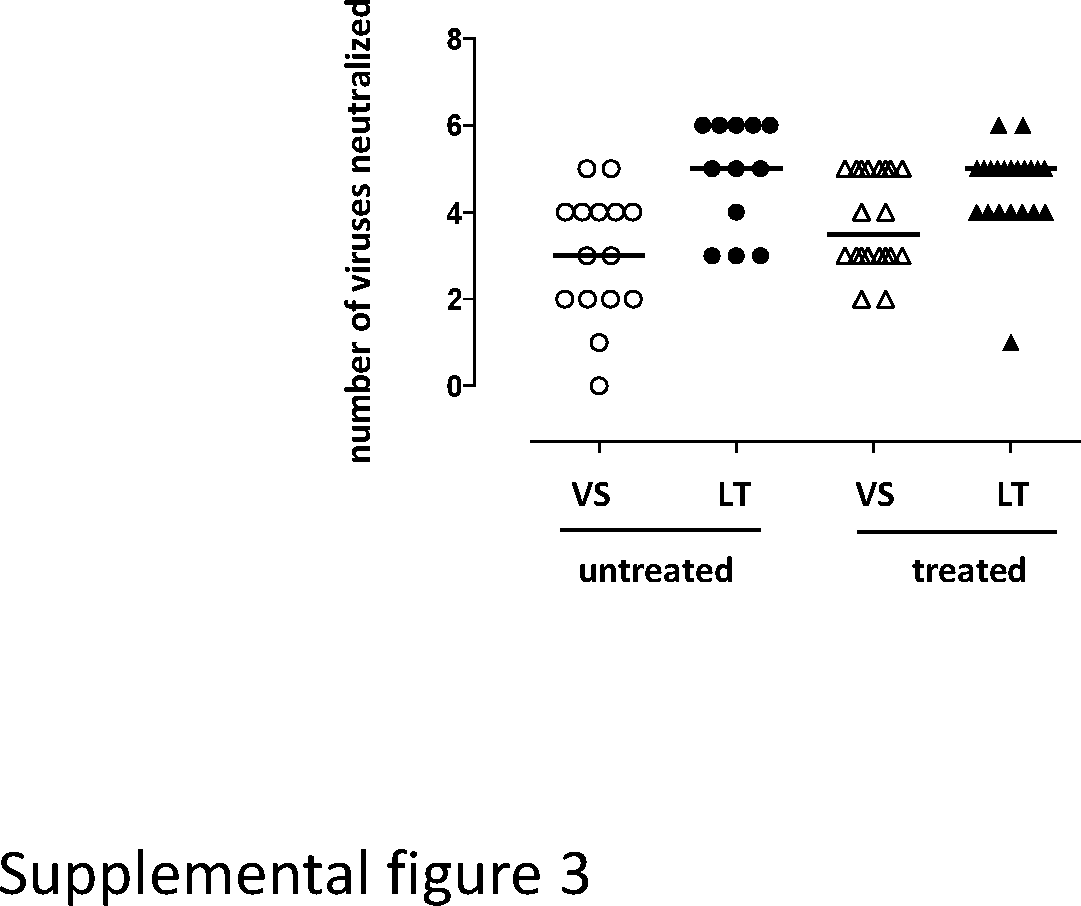

Supplement: S2 Fig — For reciprocal serum ID50 values a cutoff of 30 was used. Horizontal bars represent median values. (TIFF) [file pone.0173577.s003.tiff]
