# Supplementary material for: Longitudinal dynamics of the HIV-specific B cell response during intermittent treatment of primary HIV infection
Source: PLoS One. 2017 Mar 15;12(3):e0173577. doi: 10.1371/journal.pone.0173577 (PMC5351995; doi:10.1371/journal.pone.0173577)
Supplement: S1 Table — The direction of change is indicated by color: genes shown in red are significantly down regulated with time and genes indicated in green are significantly up regulated with time within a particular group; genes indicated in purple had significantly higher expression in the untreated group and genes indicated in blue had significantly higher expression in the treatment-interrupted group at a particular time point. P values ≤ 0.05 on the two-tailed t test were considered significant. (DOCX) [file pone.0173577.s004.docx]

| Gene | Gene expression changes significant with time in the untreated group | Gene expression changes significant with time in the treatment-interrupted group | Gene expression differences significant between the 2 groups at viral-setpoint | Gene expression differences significant between the 2 groups at the late time point | Function of protein encoded by gene |
| --- | --- | --- | --- | --- | --- |
| ABCB1 | No | No | Yes | No | ATP-binding cassette transporter |
| B4GALT3 | Yes | Yes | No | No | Synthesis of complex-type N-linked oligosaccharides in glycoproteins |
| BACH2 | Yes | No | No | Yes | Transcriptional regulator that acts as repressor or activator |
| BCL2 | Yes | No | No | No | Blocks apoptotic death of lymphocytes |
| BIN1 | Yes | Yes | No | Yes | Suppresses the cells’ transforming activity of Myc |
| BIRC3 | Yes | No | No | No | Inhibits apoptosis by binding to tumor necrosis factor receptor-associated factors TRAF1/2 |
| BIRC5 | No | Yes | No | No | Negative regulatory protein that prevents apoptotic cell death |
| BLK | Yes | No | No | Yes | B cell development, differentiation and signaling |
| CCND3 | Yes | Yes | Yes | Yes | B cell proliferation, development, and differentiation |
| CCR7 | No | Yes | No | Yes | Homing to lymph nodes |
| CD19 | Yes | Yes | Yes | Yes | Assembles with the antigen receptor of B cells lowering threshold for antigen  receptor-dependent stimulation |
| CD1c | Yes | Yes | Yes | Yes | Indicator of activation by B cell receptor signaling; mediator of EBV effects on B cells |
| CD20 | Yes | Yes | Yes | Yes | Development and differentiation of B cells into plasma cells |
| CD22 | Yes | Yes | Yes | Yes | B cell trafficking in lymphoid tissues |
| CD24 | No | Yes | Yes | No | Modulator of growth and differentiation signaling in B cells |
| CD27 | Yes | Yes | Yes | Yes | Regulates B cell activation and immunoglobulin synthesis |
| CD38 | Yes | Yes | Yes | No | Cell adhesion, signal transduction and calcium signaling |
| CD40 | Yes | No | No | No | Receptor on antigen-presenting cells that mediates immunoglobulin class switching,  memory B cell development and germinal center formation |
| CD59 | Yes | No | No | Yes | Lymphocyte signal transduction |
| CD62L | Yes | Yes | Yes | Yes | Adhesion molecule |
| CD69 | Yes | No | No | Yes | Lymphocyte proliferation and functions as a signal transmitting receptor in lymphocytes |
| CD72 | Yes | No | Yes | Yes | B cell proliferation and differentiation |
| CD83 | No | Yes | No | No | Regulation of antigen presentation |
| CD84 | Yes | Yes | Yes | Yes | Regulates receptor-mediated signaling |
| CD86 | Yes | Yes | Yes | No | Provides costimulatory signal for T cell activation and survival |
| CIITA | Yes | Yes | Yes | Yes | Positive regulator of class II major histocompatibility complex gene transcription |
| CR2 | No | Yes | No | Yes | B lymphocytes activation; EBV infection; Complement and coagulation cascades |
| CXCR5 | No | Yes | No | No | Involved in B-cell migration into B-cell follicles of spleen and Peyer patches |
| DUSP1 | Yes | Yes | No | Yes | Negative regulation of cellular proliferation |
| EBF1 | Yes | Yes | No | Yes | Transcriptional activator |
| FAS | No | No | Yes | No | Physiological regulation of programmed cell death |
| FCER2 | Yes | No | No | No | Regulation of IgE production and in the differentiation of B cells |
| FCRL1 | Yes | Yes | Yes | Yes | B-cells activation and differentiation |
| FCRL2 | Yes | Yes | Yes | Yes | Regulation of normal and neoplastic B cell development |
| FCRL3 | Yes | Yes | Yes | Yes | Potential inhibitor of B cell receptor signaling |
| FCRLA | Yes | Yes | Yes | No | Immunoglobulin assembly |
| FUT8 | Yes | Yes | Yes | Yes | Fucosyltransferase |
| GAPDH | Yes | Yes | Yes | Yes | Glycolytic enzyme |
| HSPA5 | Yes | Yes | Yes | Yes | Folding and assembly of proteins in the endoplasmatic reticulum |
| ICAM2 | Yes | No | No | Yes | Lymphocyte recirculation and immune surveillance |
| IFNA1 | No | No | Yes | No | Anti-viral activity |
| IFNAR2 | No | No | No | Yes | IFN-mediated STAT1, STAT2 and STAT3 activation |
| IFNB1 | Yes | Yes | Yes | No | Defense against viral infection |
| IFNGR1 | No | No | Yes | No | Receptor for interferon gamma |
| IGHD | Yes | Yes | No | Yes | Antigen receptor isotype delta; induces cytokine (TNF, IL1B, and IL1RN, IL6, IL10, and LIF) release |
| IGHG1 | Yes | Yes | Yes | Yes | Antigen binding, complement activation, Fc-gamma receptor signaling pathway |
| IKBKE | Yes | Yes | Yes | Yes | Regulates antiviral signaling pathways |
| IL2Ra | No | Yes | No | No | Component of low-affinity IL2 receptor |
| IL2Rb | No | Yes | No | No | Component of moderate-affinity IL2 receptor |
| IL4R | Yes | Yes | Yes | Yes | Receptor for both interleukin 4 and interleukin 13 which are involved in regulating IgE production and, chemokine and mucus production at sites of allergic inflammation |
| IL6 | No | No | No | Yes | Differentiation of B-cells into Ig-secreting cells; involved in lymphocyte and monocyte  differentiation |
| IL6R | Yes | Yes | No | No | Regulates cell growth and differentiation |
| IRF4 | Yes | Yes | No | No | Regulation of responses to viral infection |
| IRF8 | Yes | No | No | Yes | Regulates B-cell lineage specification, commitment and differentiation;  regulates peripheral tolerance by maintaining the anergic state of self-reactive B cells |
| ITGAL | Yes | No | No | Yes | Intracellular adhesion and lymphocyte costimulatory signaling |
| ITGB1 | Yes | Yes | Yes | Yes | Cell adhesion |
| ITGB7 | Yes | Yes | Yes | Yes | Adhesion molecule that mediates lymphocyte migration and homing to GALT |
| Ki67 | Yes | Yes | Yes | No | Associated with cellular proliferation |
| LRMP | Yes | Yes | Yes | Yes | Delivery of peptides to major histocompatibility complex (MHC) class I molecules |
| MAPK6 | No | No | Yes | No | Participates in protein phosphorylation cascades |
| MGAT3 | Yes | Yes | Yes | Yes | Regulation of the biosynthesis of glycoprotein oligosaccharides |
| MME | No | No | Yes | No | Peptide cleavage |
| MTA3 | Yes | No | Yes | No | Contributes to transcriptional repression by BCL6 |
| PDL1 | Yes | Yes | Yes | Yes | Immunomodulatory molecule expressed by antigen presenting cells |
| PECAM1 | Yes | No | Yes | Yes | Involved in leukocyte migration |
| POU2AF1 | Yes | Yes | Yes | Yes | Transcriptional coactivator essential for the response of B-cells to antigens and  required for the formation of germinal centers |
| PRDM1 | Yes | Yes | Yes | Yes | Drives the maturation of B-lymphocytes into Ig secreting cells |
| PTPRC | Yes | Yes | Yes | Yes | B-cell antigen receptor signaling |
| RUNX1 | No | Yes | No | Yes | Transcription factor |
| RUNX2 | No | No | Yes | No | Osteoblastic differentiation |
| SELPLG | Yes | No | Yes | No | Leukocyte trafficking during inflammation |
| SLAMF7 | Yes | No | Yes | No | Lymphocyte adhesion |
| SOX5 | Yes | Yes | Yes | No | B cell differentiation |
| SPN | Yes | Yes | Yes | Yes | Negative regulatory role in adaptive immune response |
| STAT6 | Yes | Yes | Yes | Yes | Signal transduction and activation of transcription; exerting IL4 mediated biological responses; role in class-switching |
| TNFRSF17 | Yes | Yes | Yes | No | B cell development, survival and proliferation |

S1 Table.
